# Impact of High‐Risk HPV Infection on PI3K, MALAT1, H19 and LINC00460 Expression in Cervical Cells

**DOI:** 10.1111/jcmm.70949

**Published:** 2025-11-19

**Authors:** Niloofar Neisi, Farzaneh Mousavikish, Mohammad Navid Bastani, Mehdi Parsanahad, Roya Pirmoradi

**Affiliations:** ^1^ Infectious and Tropical Diseases Research Center, Health Research Institute, Department of Medical Virology, School of Medicine Ahvaz Jundishapur University of Medical Sciences Ahvaz Iran

**Keywords:** H19, high risk, human papillomavirus, LINC00460, MALAT1, PI3K

## Abstract

High‐risk human papillomavirus (HPV) is a central factor in cervical cancer development, largely due to its E6 and E7 oncoproteins that disrupt normal cellular regulation. This study explored the influence of high‐risk HPV on the expression of PI3K and the long non‐coding RNAs (lncRNAs) MALAT1, H19 and LINC00460 in cervical cells. Using a case–control design, cervical liquid samples from 50 HPV‐positive patients and 20 healthy controls were analysed via quantitative real‐time PCR, with statistical methods employed to assess correlations between viral oncoproteins and target gene expression. Results demonstrated a significant upregulation of PI3K (24.59‐fold change, *p* < 0.036), MALAT1 (9.75‐fold change, *p* < 0.005), LINC00460 (1.15‐fold change, *p* < 0.013) and H19 (7.1‐fold change, *p* < 0.018) in HPV‐infected samples, indicating their potential role in HPV‐mediated oncogenesis. Although correlation analysis revealed trends between E6/E7 and certain lncRNAs, these were not statistically significant. Overall, these findings deepen our understanding of the molecular changes linked to high‐risk HPV infections and identify PI3K, MALAT1 and H19 as promising biomarkers and therapeutic targets for cervical cancer. Future studies should further investigate these interactions to enhance early detection and improve treatment strategies for HPV‐associated malignancies.

## 1 Introduction

Cervical cancer is a major public health concern, representing one of the most prevalent and lethal malignancies of the female reproductive system worldwide [1]. The pioneering work of zur Hausen established that Human Papillomavirus (HPV) infection—especially by high‐risk types such as HPV‐16 and HPV‐18—is a critical etiological factor in cervical carcinogenesis [2, 3]. HPV integration into host cervical cells induces genetic and cellular alterations primarily through the action of viral oncoproteins E6 and E7, which inactivate key tumour suppressor proteins (p53 and Rb) and facilitate malignant transformation [4].

HPV is a double‐stranded, non‐enveloped DNA virus with a genome organised into early (E), late (L) and long control regions (LCR) [5]. The early genes, including E6 and E7, are essential for viral replication and oncogenic transformation [6, 7], while the late genes encode structural proteins such as L1, which is a target for current immunisation strategies [8, 9]. With over 200 distinct types, HPV is classified into high‐risk and low‐risk groups based on its oncogenic potential [10, 11].

### 1.1 PI3K/Akt Pathway and Its Oncogenic Implications

The PI3K/Akt signalling pathway is fundamental for regulating cellular processes such as proliferation, survival, metabolism and migration. Activation of this pathway begins with the conversion of phosphatidylinositol‐4,5‐bisphosphate (PIP2) into phosphatidylinositol‐3,4,5‐trisphosphate (PIP3), which in turn recruits and activates the serine/threonine kinase Akt [12, 13]. Once activated, Akt phosphorylates a wide array of downstream targets, including the mammalian target of rapamycin (mTOR), thereby promoting protein synthesis, cell growth and survival [14]. Dysregulation of the PI3K/Akt/mTOR axis—frequently due to genetic alterations in components such as PIK3CA, PTEN, Akt and mTOR—is a common event in cervical cancer and contributes to tumour progression, metastasis and resistance to apoptosis. Consequently, therapeutic strategies targeting this pathway, including various PI3K inhibitors, are being actively explored in clinical trials [15].

### 1.2 Role of Long Non‐Coding RNAs in HPV‐Associated Carcinogenesis

In parallel with the PI3K/Akt pathway, long non‐coding RNAs (lncRNAs) have emerged as pivotal regulators of gene expression in cancer. LncRNAs, defined as RNA molecules exceeding 200 nucleotides in length that do not encode proteins, modulate gene expression through diverse mechanisms such as chromatin remodelling, transcriptional control and post‐transcriptional regulation [16, 17, 18]. Among these, MALAT1, H19 and LINC00460 have gained significant attention for their roles in oncogenesis.

MALAT1: A highly conserved lncRNA, MALAT1 is implicated in tumour progression through its ability to regulate cell proliferation, migration and epithelial‐to‐mesenchymal transition (EMT). Its overexpression in various cancers, including cervical cancer, correlates with poor prognosis and enhanced metastatic potential [19, 20, 21]. MALAT1 exerts its effects by interacting with critical signalling pathways, notably PI3K/Akt and Wnt/β‐catenin, and by acting as a molecular sponge for tumour‐suppressive microRNAs [22, 23, 24]. H19: Typically expressed during embryogenesis and silenced in adult tissues, H19 is reactivated in multiple malignancies where it functions as an oncogenic driver. In cervical cancer, H19 contributes to increased cell proliferation, migration and resistance to chemotherapy. Its dual role—as both an oncogene and a tumour suppressor, depending on the context—highlights its complex involvement in tumour biology and underscores its potential as a biomarker for disease progression [17, 25]. LINC00460: Recently identified as a significant regulator in various cancers, LINC00460 is generally associated with aggressive tumour phenotypes, including larger tumour size, advanced clinical stage, lymph node metastasis and reduced overall survival. Acting as a competitive endogenous RNA (ceRNA), LINC00460 modulates the activity of multiple oncogenic pathways by sequestering microRNAs. Interestingly, our findings of LINC00460 in HPV‐positive cervical cells suggest that its role may be uniquely modulated by HPV oncoproteins, indicating a complex regulatory network that merits further investigation [26, 27].

### 1.3 HPV Oncoproteins and Their Influence on Gene Expression

HPV oncoproteins E6 and E7 are central to the virus's ability to induce carcinogenesis. By targeting tumour suppressor proteins such as p53 and Rb, these oncoproteins not only disrupt cell cycle regulation and apoptosis but may also influence the expression of key cellular genes, including those involved in the PI3K/Akt pathway and lncRNA‐mediated regulation. While the oncogenic potential of E6 and E7 is well established, their precise impact on the expression of PI3K, MALAT1, H19 and LINC00460 remains to be fully elucidated. Despite significant advances in our understanding of HPV‐induced carcinogenesis, the specific interactions between high‐risk HPV infections and the expression of critical oncogenic regulators such as PI3K, MALAT1, H19 and LINC00460 are not completely understood. Moreover, the influence of these genes on the expression of viral oncoproteins E6 and E7, which are pivotal in driving cancer progression, has not been thoroughly investigated in clinical settings.

This study aims to address these gaps by systematically examining the impact of high‐risk HPV infections on the expression profiles of PI3K, MALAT1, H19 and LINC00460 in cervical samples compared to a control group. By elucidating the molecular interplay between HPV oncoproteins and these regulatory genes, our research seeks to provide deeper insights into the mechanisms underlying HPV‐induced cervical cancer. Ultimately, this work may identify novel biomarkers and therapeutic targets, thereby contributing to the development of improved strategies for early detection, prognosis and treatment of cervical cancer.

## 2 Materials and Methods

### 2.1 Study Population

In this case control study, a total of 50 liquid samples were collected from patients diagnosed with high‐risk human papillomavirus (HPV). These samples were obtained through biopsies performed by a gynaecologist. The presence of high‐risk HPV types in these patients was confirmed through quantitative real‐time PCR (qRT‐PCR) testing, which was conducted at the Keyvan Laboratory in Tehran. Additionally, a control group consisting of 20 healthy individuals was also included, with their samples being tested by real‐time PCR to confirm the absence of HPV infection. These samples were used as a negative control group to compare the results with those from the HPV‐infected patients. Patients with other infectious diseases of the genital tract were excluded from the study. Written informed consent was obtained from all participants (Table 1). All stages of the study were approved by the Ethics Committee of Ahvaz Jundishapur University of Medical Sciences (IR.AJUMS.MEDICINE.REC.1401.082).

**TABLE 1:** Characteristics of patient and control groups.

Parameter	Patient group (HPV positive)	Control group (HPV negative)
Number of patients	50	20
Age (mean ± SD)	43.76 ± 10.43	42.52 ± 9.26
Age range	25–65	25–58
Gender	Female	Female
HPV high risk genotypes	16 (28%), 31 (20%), 52 (20%), 51 (14%), 58 (12%), 59 (12%), 56 (10%), 18 (8%), 45 (8%), 68 (8%), 35 (6%), 39 (4%)	—
Smoking status	—	—

*Note:* Age (year), Gender (female/male), HPV high risk genotypes (prevalence of HPV high risk Genotype in patients, %).

### 2.2 Sample Preparation and RNA Extraction From Liquid Samples

The collected samples were immediately transported to the virology laboratory under controlled cold chain conditions to maintain the integrity of the samples. Once at the laboratory, the liquid samples were processed by centrifuging at 3000 RPM for 5 min to separate the cellular components from the liquid phase. The cellular pellet, which contains the nucleic acids of interest, was carefully collected and stored at −20°C to prevent RNA degradation and ensure the preservation of genetic material for further analysis. Liquid samples from both patient and control groups were processed using the Super RNA Extraction Kit (Ana Cell, Iran) according to the manufacturer's protocol. The procedure involved cell lysis, purification, and contaminant removal to obtain high‐quality total RNA, which was subsequently quantified to ensure adequate yield and integrity for downstream applications. To guarantee high‐quality RNA, we conducted spectrophotometric analysis, which verified an A260/A280 ratio of approximately 2.1, suggesting low levels of protein contamination. Furthermore, we evaluated RNA integrity through agarose gel electrophoresis/Bioanalyzer, revealing clear rRNA bands with no notable degradation.

### 2.3 cDNA Synthesis

Extracted RNA was converted into complementary DNA (cDNA) using the Ana Cell cDNA synthesis kit (Iran). This reverse transcription process employed a reverse transcriptase enzyme, derived from RNA viruses, to synthesise cDNA from the RNA template, enabling subsequent quantification by real‐time PCR. In summary, 1000 ng of extracted RNA was mixed with 0.5 μL of random hexamer and 0.5 μL of Oligo dT (for targeting poly A tail of mRNA). Following a 5‐min incubation at 70°C, a mixture of buffer RT, dNTP mix, and RT enzyme was added and incubated for 60 min at 42°C. The reaction was then heated at 70°C for 5 min. The resulting cDNA was stored at −20°C.

### 2.4 Real‐Time PCR

Quantitative real‐time PCR was conducted to assess the expression levels of target genes. Reactions were prepared in a total volume of 20 μL containing cDNA template, gene‐specific primers and SYBR Green master mix (Table 2). Thermal cycling was performed on an appropriate real‐time PCR system with the following conditions: an initial denaturation at 95°C for 10 min, followed by 40 cycles of denaturation at 95°C for 15 s and annealing/extension at 60°C for 1 min. Fluorescence was measured at the end of each cycle, Efficiency Calculation: Efficiency (E) is calculated by preparing serial dilutions of cDNA, followed by PCR using GAPDH primers as a reference. A standard curve is created, and E and *R* ^2^ values are calculated. Acceptable values for E are > 95%, and *R* ^2^ should be close to 1. The relative expression levels were determined using the 2^−ΔΔCt^ approach [28]. Validation of Results: Melting Curve: Used to verify the specificity of the PCR products. Each gene has a specific melting curve, and all samples should show a single peak. Agarose Gel Electrophoresis: PCR products are visualised on a 2% agarose gel after electrophoresis, comparing the bands to a DNA ladder to confirm the presence of the correct amplicon.

**TABLE 2:** Characteristics of primers used for RT‐PCR.

Primer name	Primer sequence
PI3K	5′‐GGTTGTCTGTCAATCGGTGACTGT‐3′ 5′‐GAACTGCAGTGCACCTTTCAAGC‐3′
MALAT1	5′‐CTTCCCTAGGGGATTTCAGG‐3′ 5′‐GCCCACAGGAACAAGTCCTA‐3′ [29]
H19	5′‐ACTCAGGAATCGGCTCTGGAAG‐3′ 5′‐GCTGCTGTTCCGATGGTGTC‐3′ [30]
LINC00460	5′‐GGATG AACCA CCATT GCC‐3′ 5′‐CCCAC GCTCA GTCTT TCT‐3′ [31]
GAPDH	5′‐CGACCACTTTGTCAAGCTCA‐3′ 5′‐CCCTGTTGCTGTAGCCAAAT‐3′
E6/E7	5′‐ATGCATGGACCTAAGGCAAC‐3′ 5′‐AGGTCGTCTGCTGAGCTTTC‐3′ [32]

#### 2.4.1 Statistical Analysis

Gene expression data were analysed using the Relative Expression Software Tool (REST), which applies a pair‐wise fixed reallocation randomisation test to assess the statistical significance of expression differences between control and experimental groups. Ct values for target and reference genes were imported into the software, and PCR efficiencies were either provided or assumed to be 100% (efficiency = 2.0) when unknown. REST calculates relative expression ratios based on efficiency‐corrected ΔΔCt values and performs 2000 random reallocations of samples to generate *p*‐values. This non‐parametric approach does not assume normal distribution and is particularly suited for small sample sizes and real‐time PCR data.

Data were analysed using descriptive statistics, including the median and interquartile range (IQR), to account for non‐normal distribution and reduce the influence of outliers. The IQR was calculated as the difference between the 75th percentile (Q3) and the 25th percentile (Q1). For group comparisons, non‐parametric tests were employed where appropriate, specifically the Mann–Whitney *U* test for assessing differences between independent groups. Outliers were identified using the standard IQR method, defined as values below Q1–1.5 × IQR or above Q3 + 1.5 × IQR.

Spearman's rank correlation analysis was conducted using IBM SPSS Statistics (version 24) to assess the association between study variables. This non‐parametric method was selected due to the non‐normal distribution of data, as determined by the Shapiro–Wilk test. Correlation strength was interpreted based on Spearman's rho (*ρ*) values ranging from 0 to ±1, with higher absolute values indicating stronger associations. Statistical significance was determined using a *p*‐value threshold of < 0.05.

## 3 Results

The study's findings reveal that the expression levels of the analysed genes were markedly elevated in individuals infected with high‐risk HPV compared to the control group. Specifically, the expression of MALAT1 (*p* < 0.005), PI3K (*p* < 0.036), H19 (*p* < 0.018) and LINC00460 (*p* < 0.013) was significantly higher in the HPV‐positive cohort (Table 3).

**TABLE 3:** mRNA expression in control and patient groups.

Gene	Control group median (IQR)	Patients group median (IQR)	Fold changes	*p*
H19	5.80 (3.69–8.15)	8.39 (6.37–9.4)	7.1	0.018
MALAT1	5.02 (3.5–6.07)	7.6 (3.06–12.13)	9.75	0.005
LINC00460	5.5 (4.70–7.95)	8.96 (5.72–10.49)	1.15	0.013
PI3K	5.54 (1.26–6.30)	6.62 (4.99–8.66)	24.59	0.036

**FIGURE 1:**
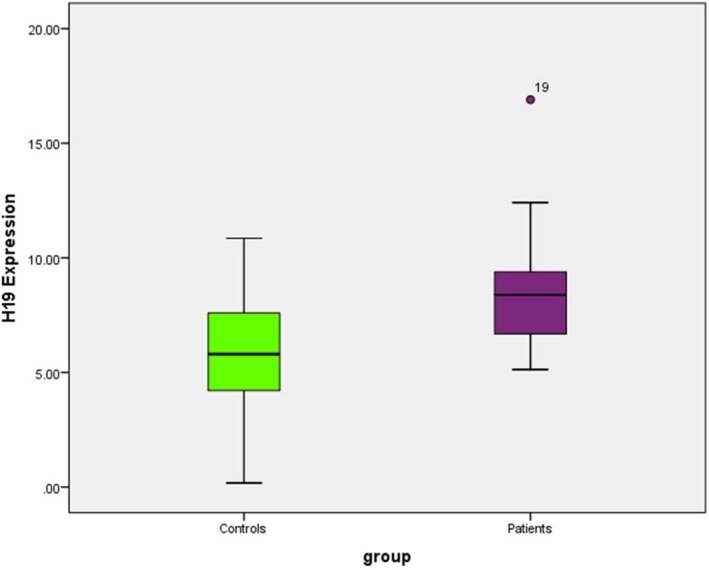
Difference in lncRNA H19 expression between patients and the control group.

H19 expression levels were significantly higher in patients compared to controls (*U* = 131.000, *Z* = −2.375, *p* = 0.018, Mann–Whitney *U* test). Median expression in patients was 8.39 (IQR: 6.37–9.40), while controls showed a median of 5.80 (IQR: 3.69–8.15) (Figure 1).

**FIGURE 2:**
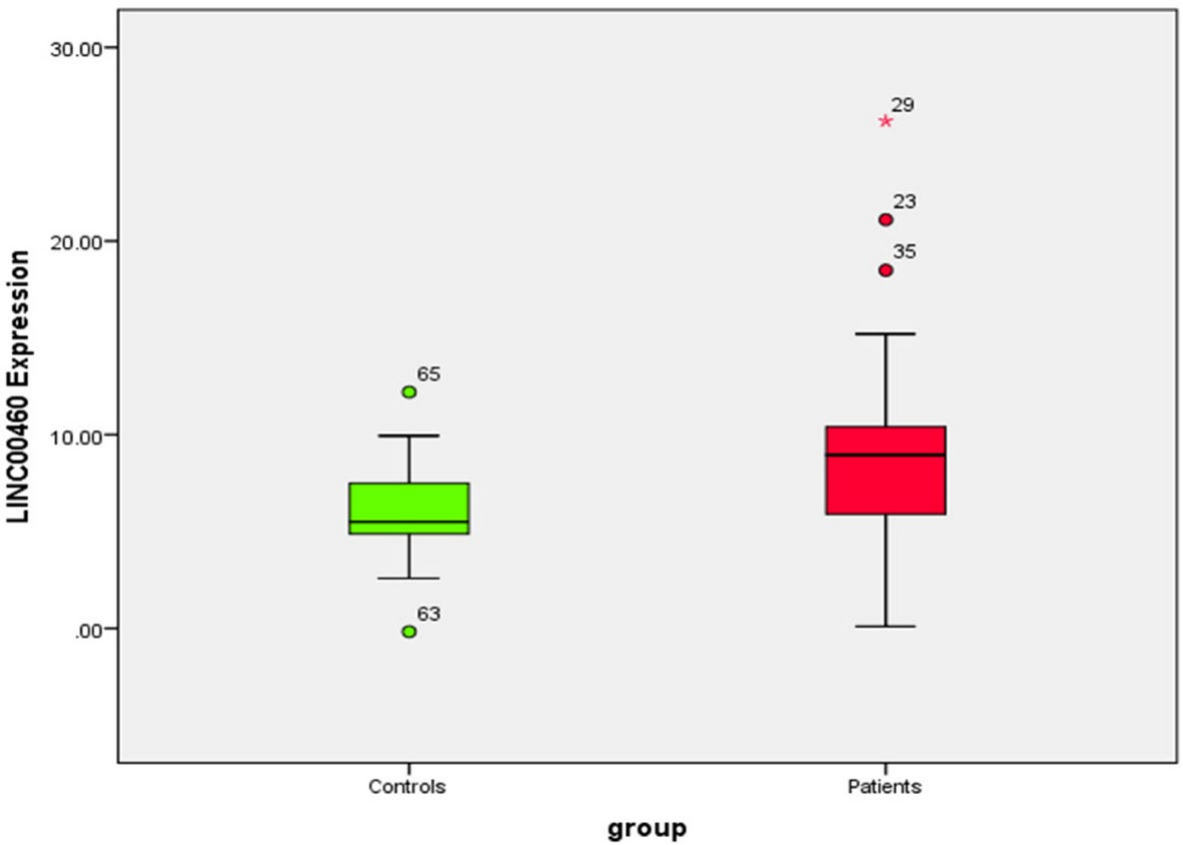
Difference in lncRNA LINC00460 expression between patients and the control group.

LINC00460 expression was significantly higher in patients compared to controls, as determined by the Mann–Whitney *U* test (*U* = 252.000, *Z* = −2.493, *p* = 0.013, two‐tailed). Median expression in the patient group was 8.96 (IQR: 5.72–10.49), while the control group had a median of 5.50 (IQR: 4.70–7.95), indicating elevated expression in patients (Figure 2).

**FIGURE 3:**
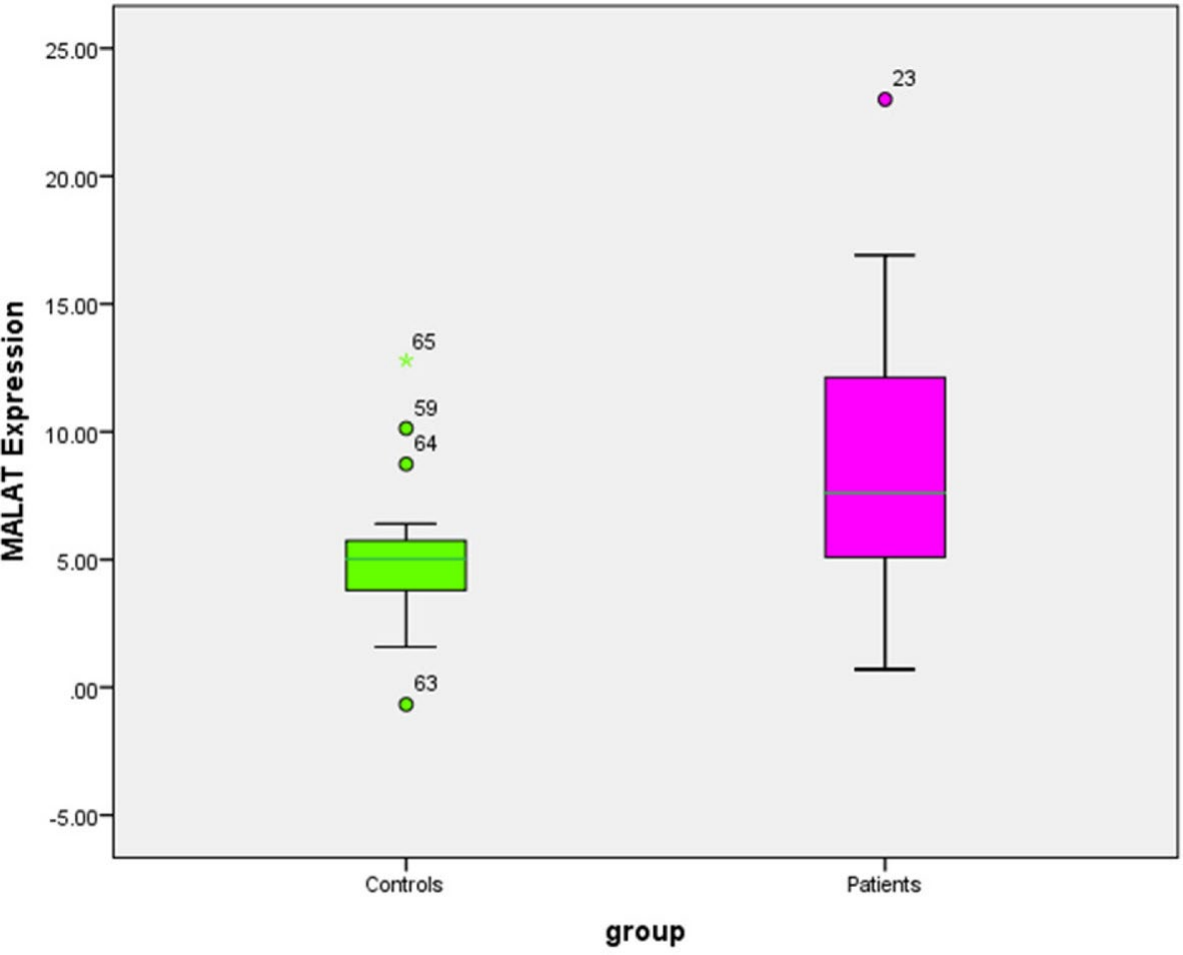
Difference in lncRNA MALAT1 expression between patients and the control group.

MALAT1 expression was significantly higher in patients compared to controls, as assessed by the Mann–Whitney *U* test (*U* = 230.500, *Z* = −2.803, *p* = 0.005, two‐tailed). The median expression level in the patient group was 7.60 (IQR: 3.06–12.13), while the control group had a median of 5.02 (IQR: 3.50–6.07), indicating elevated MALAT1 expression in patients (Figure 3).

**FIGURE 4:**
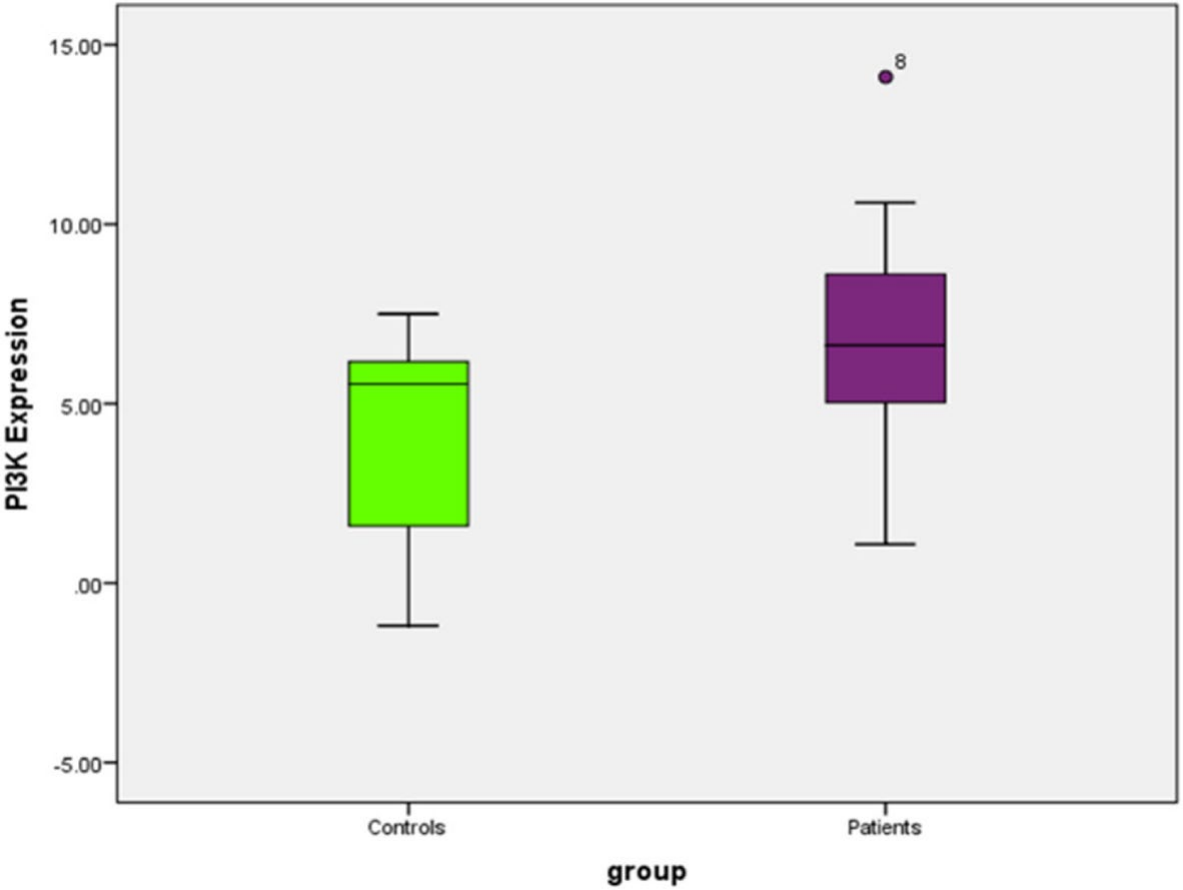
Difference in PI3K expression between patients and the control group.

**FIGURE 5:**
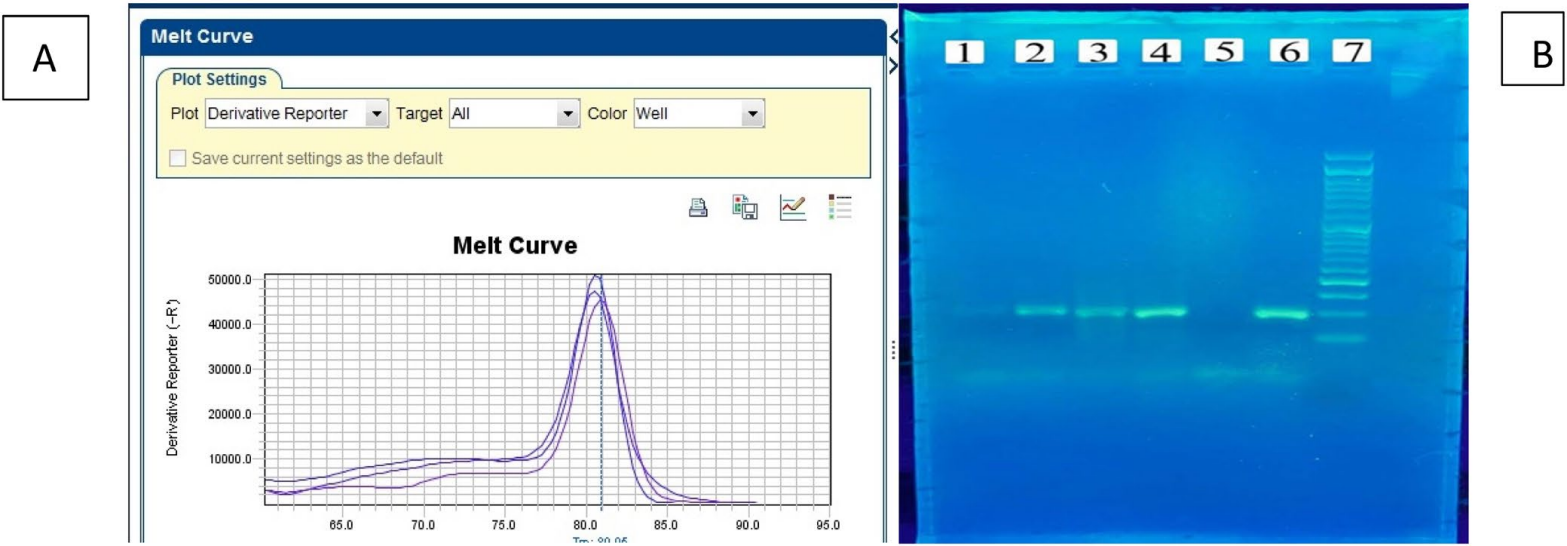
(A) PI3K melting curve; (B) 2% agarose gel electrophoresis of PI3K. Lines 1, 2, 3, 4 and 6 positive PI3K samples, 108 bp. Line 5 NTC sample. Line 7 ladder 50 bp (Sinaclone, Iran).

PI3K expression was significantly higher in patients compared to controls, as determined by the Mann–Whitney *U* test (*U* = 97.000, *Z* = −2.096, *p* = 0.036, two‐tailed). The median expression in the patient group was 6.62 (IQR: 4.99–8.66), while the control group had a median of 5.54 (IQR: 1.26–6.30), indicating elevated PI3K expression in patients (Figures 4 and 5).

A positive correlation was observed between the E6/E7 genes of HPV and the genes MALAT1, PI3K and H19. These correlations suggest that the E6 and E7 proteins, which are HPV oncoproteins, may directly or indirectly regulate or activate these genes, playing a significant role in the process of carcinogenesis. This finding aligns with our data and demonstrated an increase in LINC00460 in patients who are positive for high‐risk HPV.

**FIGURE 6:**
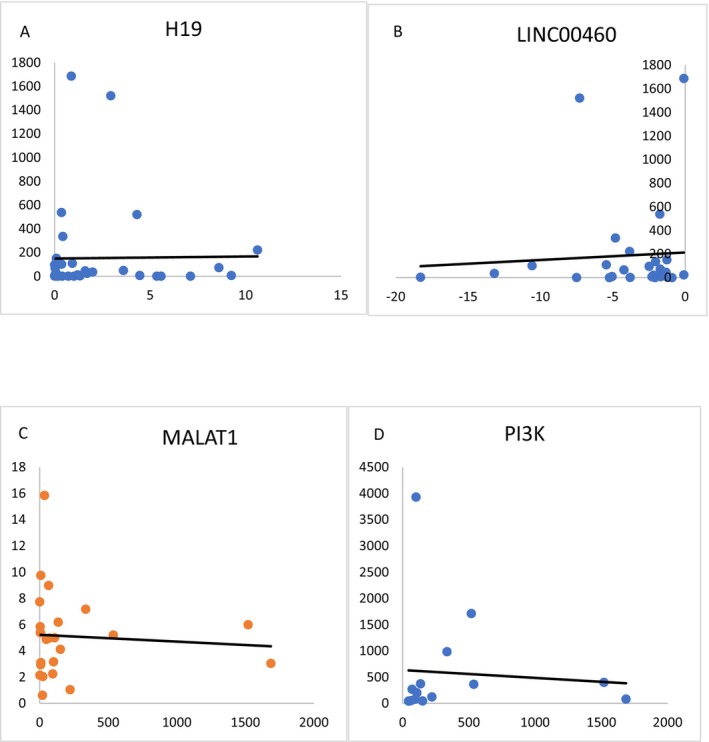
Correlation between (A) lncRNA H19 and E6/E7 expressions, (B) lncRNA LINC00460 and E6/E7 expressions, (C) MALAT1 and E6/E7 expressions and (D) PI3K and E6/E7 expressions.

There was a positive correlation between the expression of H19 and E6/E7, which was not statistically significant (rho = 0.02, *p* < 0.89). There was a negative correlation between the expression of LINC00460 and E6/E7, which was not statistically significant (rho = −0.02, *p* < 0.9). There was a positive correlation between the expression of MALAT1 and E6/E7, which was not statistically significant (rho = 0.07, *p* < 0.75). There was a positive correlation between the expression of PI3K and E6/E7, which was not statistically significant (rho = 0.04, *p* < 0.88) (Figure 6, Table 4).

**TABLE 4:** Pearson's rho (p) correlation coefficients between E6/E7 and selected lncRNAs (H19, MALAT1 and LINC00460), PI3K, and among each other.

Gene	H19	MALAT1	LINC00460	PI3K	E6/E7
H19	1.00	0.16	−0.19	0.14	0.02
MALAT1	0.16	1.00	−0.28	−0.13	0.07
LINC00460	−0.019	−0.28	1.00	−0.429	−0.02
PI3K	0.147	−0.134	−0.429	1.00	0.04
E6/E7	0.255	0.050	−0.39	0.04	1.00

*Note:* The green highlight indicates the column showing correlations between E6/E7 and other genes. Diagonal values of 1.00 indicate self correlation. No statistical significance is implied.

## 4 Discussion

This study investigated the relationship between high‐risk Human Papillomavirus (HPV) infections and the expression of PI3K, MALAT1, H19 and LINC00460 in cervical cells compared to a control group. Our findings revealed significant alterations in the expression levels of these genes in HPV‐positive samples, offering insights into potential mechanisms by which HPV—especially through its oncoproteins E6 and E7—contributes to cervical carcinogenesis. This study reveals an upregulation of PI3K and MALAT1 in HPV‐infected cervical cells relative to healthy controls. This observation is consistent with previous reports, such as those by Henken et al. (2011) [33], which demonstrated increased PIK3CA expression in HPV‐positive cervical cancer cells, implicating activation of the PI3K/AKT pathway in HPV‐driven tumorigenesis. Similarly, studies by Jiang (2014) [34] and Sun (2020) [35] have highlighted the role of elevated MALAT1 expression in various cancers, including cervical cancer, where it appears to promote proliferation, invasion, and angiogenesis of cervical cancer cells by serving as a precursor for microRNA (miRNA) or as a sponge for miRNA, thus impacting the post‐transcriptional regulation of gene expression [36]. Our data reinforce these findings, suggesting that MALAT1 is actively involved in HPV‐induced cancer progression. Notably, this study identified a novel observation: The negative correlation between LINC00460 and E6/E7 expression indicates a complex regulatory interplay among lncRNAs during HPV‐mediated oncogenesis, warranting further investigation.

The positive correlation between E6/E7 expression and the upregulation of PI3K and MALAT1 supports the hypothesis that HPV oncoproteins modulate these genes either directly or indirectly. E6 and E7 are known to interfere with key cellular processes by inactivating tumour suppressor proteins like p53 and Rb, thereby disrupting apoptosis, cell cycle control and immune responses. These findings suggest that the upregulation of PI3K and MALAT1 forms part of the molecular network influenced by these viral proteins during carcinogenesis. These results are in line with earlier studies. Jiang (2014) [34] and Sun (2020) [35] demonstrated that HPV infection, via activation of the PI3K/AKT pathway, leads to increased MALAT1 expression. Hao (2020) [37] further supported this concept by showing that HPV18 E6/E7 proteins can induce MALAT1 expression through the IL‐6/STAT3 pathway. Additionally, our findings regarding H19 expression concur with László et al. (2021) [38], who reported alterations in several lncRNAs, including H19, in HPV‐positive cervical cancer cells. Together, these studies highlight the significant role of lncRNAs in HPV‐mediated oncogenesis.

Numerous studies have highlighted the involvement of LINC00460 in the development of various cancers, yet its function in cervical cancer remains uncertain. Fan Li demonstrated that LINC00460 can promote angiogenesis through the activation of NF‐κB [39]. A different study indicated that LINC00460 encourages the growth of cervical cancer cells and prevents apoptosis through the sponging of miR‐503‐5p [40]. These studies are consistent with our data and revealed upregulation of LINC00460 in high‐risk HPV positive patients.

Our research uncovers a new negative regulatory relationship involving LINC00460 and the expression of HPV E6/E7 in cervical cancer cells that are HPV‐positive, which is different from the known oncogenic function of LINC00460. The increased expression of LINC00460 in HeLa and SiHa cell lines led to a significant decrease in both E6/E7 mRNA and protein levels, which is associated with a restoration of p53/pRb activity and a reduction in cell proliferation [41]. We propose that LINC00460 could function as a ceRNA, soaking up miRNAs that help stabilise E6/E7 transcripts, or may attract epigenetic repressors to inhibit E6/E7 transcription [42]. This discovery corresponds with the tumour‐suppressive functions of lncRNAs such as DINO in HPV‐positive cells [17] but contradicts the prevailing perception of LINC00460 as an oncogene. The negative regulation might indicate a role that is specific to the context during the initial stages of HPV infection, potentially restricting E6/E7‐driven oncogenesis until viral integration disrupts this balance. In a clinical setting, LINC00460 may act as a biomarker for early‐stage lesions or a target for therapies aimed at inhibiting E6/E7 activity. Future research ought to investigate the mechanisms at play, such as the interaction of LINC00460 with miRNAs or epigenetic complexes, and confirm these results in vivo and among various HPV types.

While this study provides valuable insights, certain limitations must be acknowledged. The sample size was relatively small (50 HPV‐positive patients and 20 healthy controls), which may limit the generalizability of our results. Moreover, the observational design of this study means that the results are correlative, and additional functional studies are needed to elucidate the precise mechanistic roles of these lncRNAs and their interactions with HPV oncogenes. Future research should aim to expand the sample size and include diverse populations to validate and extend these findings. Functional studies using RNA interference or CRISPR technology could further clarify the roles of PI3K, MALAT1, H19 and LINC00460 in HPV‐mediated carcinogenesis. Moreover, exploring the dynamic interplay between E6/E7 and these lncRNAs could offer deeper insights into the molecular mechanisms underpinning HPV‐associated cancers. Finally, longitudinal studies are recommended to evaluate the potential of these genes as biomarkers for early detection and prognostication in cervical cancer.

## 5 Conclusion

In conclusion, this study identifies altered expression patterns of PI3K, MALAT1, H19 and LINC00460 in HPV‐positive cervical cells. While these changes suggest potential involvement in HPV‐associated carcinogenic processes, further validation is needed to confirm their roles. Preliminary trends observed between E6/E7 expression and these genes may indicate possible regulatory interactions, though these correlations were not statistically significant. Overall, our findings contribute to the growing understanding of HPV‐related molecular changes and offer a foundation for future research into potential biomarkers and therapeutic targets in cervical cancer.

## Author Contributions

**Niloofar Neisi:** conceptualization (equal), data curation (equal), funding acquisition (lead), investigation (equal), methodology (equal), project administration (equal), supervision (equal), validation (equal), visualization (equal), writing – review and editing (equal). **Farzaneh Mousavikish:** investigation (equal), methodology (equal), project administration (equal), writing – original draft (equal). **Mohammad Navid Bastani:** data curation (equal), methodology (equal), writing – original draft (equal), writing – review and editing (equal). **Mehdi Parsanahad:** conceptualization (equal), methodology (equal), validation (equal). **Roya Pirmoradi:** investigation (equal), methodology (equal), visualization (equal).

## Ethics Statement

This study was approved by the Ethics Committee of Jundishapur University of Medical Sciences, Ahvaz, Iran, and the ethical number is (IR.AJUMS.MEDICINE.REC.1401.082).

## Conflicts of Interest

We declare that we have no conflicts of interest to disclose. We are not associated with any commercial entity, and there are no financial or non‐financial interests that could influence the objectivity of the scientific content presented in this article.

## Data Availability

The majority of the data supporting the conclusions of this study can be obtained upon request from the corresponding author. However, certain data are not publicly accessible due to restrictions, such as containing information that may compromise the privacy of research participants.
